# Development of Chinese Junior High School Students’ Creative Potential: Within-Person and Between-Person Effects of Student–Student Support and Need for Cognition

**DOI:** 10.3389/fpsyg.2020.552831

**Published:** 2020-10-08

**Authors:** Peipei Chen, Jinghuan Zhang

**Affiliations:** Department of Psychology, Shandong Normal University, Jinan, China

**Keywords:** student–student support, need for cognition, creative potential, Chinese junior high school students, the within-person and between-person effects

## Abstract

A longitudinal study was conducted to examine the developmental trend of creative potential in Chinese junior high school students and the within-person and between-person effects of student–student support and need for cognition. Two hundred and fourteen Chinese junior high school students participated in the present study (mean age = 13.29 years, SD = 0.49 years, 116 boys). Student–student support, need for cognition, and creative potential were measured once per year for 3 years. Longitudinal multilevel models indicated that (1) Chinese junior high school students’ creative potential showed a downward trend from grades 7 to 9; (2) at the within-person level, time-varying student–student support positively predicted time-varying creative potential; (3) at the within-person level, time-varying need for cognition moderated the positive link between time-varying student–student support and time-varying creative potential; and (4) at the between-person level, no support was found for the links between student–student support, need for cognition, and creative potential. Specifically, average levels of student–student support neither significantly predicted initial levels and developmental rates of creative potential nor moderated the links between average levels of student–student and initial levels and developmental rates of creative potential. The findings highlight that at the within-person and between-person levels, student–student support and need for cognition have differential influences on Chinese junior school students’ creative potential.

## Introduction

In the 21st-century knowledge society, information bases rapidly change and grow. Consequently, it has never been more important to develop creativity to face unknown challenges of the future (Amabile, 1996; Kleibeuker et al., 2016). Creativity refers to the ability to produce a product characterized by novelty and usefulness (Sternberg and Lubart, 1999; Plucker et al., 2004). For adolescents, creativity is beneficial to solving daily problems (Runco, 2004), promotes academic performance (Gajda et al., 2017), helps maintain high levels of well-being (Tamannaeifar and Motaghedifard, 2014), enables good interpersonal relationships (Sagone and Caroli, 2013), and protects from experiencing poor emotional problems (Schuldberg, 2001; Byron and Khazanchi, 2011). Moreover, adolescents who demonstrate creativity can win competitions and resolve conflicts (Lassig, 2012), address mounting social and ecological issues in our global society (Craft, 2011), and obtain good career opportunities (Milgram and Hong, 1993). For these reasons, creativity has been regarded as a central ability needed by adolescents to survive and achieve success in competitive and global surroundings (Florida, 2002). Given the extremely important role of creativity in adolescents, it is necessary to figure out what the developmental trend of adolescents’ creative potential is and what factors affect the development of creative potential. In all stages of adolescence, the junior high school stage is a crucial phase because during this stage, students transit from childhood to adolescence and significant shifts in individuals’ physical and psychological characteristics take place (Erikson, 1966). Thus, the present study focused especially on the developmental trend of creative potential and its influencing factors in junior high school students.

In the area of creativity research, various measurements have been adopted to distinguish people with high and low creativity. Even if one’s divergent thinking ability is not equal to one’s creativity, divergent thinking ability is still one of the most widely used effective creative potential indicators (Charles and Runco, 2001; Barbot et al., 2016). Divergent thinking ability refers to the ability to generate many diverse creative ideas (Guilford, 1956). Thus, in the present study, creative potential was indicated by divergent thinking ability.

### Developmental Trends of Creative Potential in Junior High School Students

By employing divergent thinking ability as the indicator of creative potential, several attempts have been made to explore the developmental trend of creative potential among junior high school students. For example, Cheung et al. (2004) found that there existed a growth trend in students’ creative potential from grades 7 to 9 (ages 12 to 14). However, some studies showed a significant decrease of creative potential in junior high school students. For example, a longitudinal investigation reported that an obvious decline occurred on adolescents’ creative potential from grades 6 to 9 (ages 11 to 14; Camp, 1994). Similarly, Yi et al. (2013) showed a general decline of creative potential for Chinese students from grades 7 to 9 (ages 12 to 14). Besides these studies documenting the increase and decrease of creative potential in junior high school students, some studies reported few age differences in creative potential. For example, Claxton et al. (2005) performed a longitudinal study and found that the developmental process of students’ creative potential remained stable from grades 6 to 9 (ages 11 to 14). Along the same lines, in the study of Kleibeuker et al. (2013), no age group differences between grades 7/8 and 10/11 (12-/13-year-olds and 15-/16-year-olds) were observed for fluency, flexibility, and originality of the verbal divergent thinking.

Although the studies mentioned above provided a large amount of information about the developmental trend of creative potential among junior high school students, most of these studies were cross-sectional, and thus, the validity of the findings of these studies may not be guaranteed since it is difficult to untangle cohort effects from actual developmental effects (Baltes and Reinert, 1969; Murnane and Willett, 2010). Even if very limited studies adopted longitudinal design, such as Claxton et al. (2005) and Camp (1994), several important issues remained unaddressed. First, these longitudinal studies suffered from small sample sizes (*n* = 33 and *n* = 25), which limited the generalization of the conclusions (Seddon and Scheepers, 2012). Second, the method of repeated-measures analysis of variance was used to analyze the developmental trend of creative potential in these longitudinal studies. This method should require the following assumptions: (a) variance is homogeneous and (b) random errors are independent of each other. However, these assumptions are rarely met in longitudinal studies, and thus, the adoption of this method might result in unreasonable or even erroneous conclusions (Shin, 2009; Chandrasekhar, 2011). The emerging longitudinal data analysis methods, such as multilevel linear models, do not require longitudinal samples to meet these assumptions and are more suitable for longitudinal research (Shin, 2009). Third, the two longitudinal studies were conducted in Western background. As Yi et al. (2013) found, the developmental trajectories of junior high school students’ creative potential between Western and Eastern cultures can be distinct. This is because teachers’ attitudes toward students and students’ own academic pressure which have direct impacts on the development of students’ creative potential are not the same or even very different in the two cultures (Yi et al., 2013). Therefore, it is necessary to examine the developmental trend of creative potential in Eastern junior high school students such as Chinese students. Taking these factors into consideration, the first purpose of the present study was to examine the developmental trend of creative potential in Chinese junior high school students by using multilevel linear models with an adequate sample.

### Student–Student Support and Creative Potential

Student–student support is defined as perceived emotional support among students (i.e., fraternal loving and respecting each other; Bachman and O’Malley, 1986; Jia et al., 2009). According to ecological theories of development, students who perceive benign school environments, such as student–student support, will experience a “match” between their developmental needs and the environment which they are embedded in and consequently experience positive developmental outcomes (Bronfenbrenner, 1979). Findings from previous research are also consistent with the theory, showing that student–student support was related to higher concurrent and prospective self-esteem (Colarossi and Eccles, 2003; Loukas et al., 2006; Way et al., 2007), higher academic adjustment (Danielsen et al., 2010; Wentzel et al., 2010), and less depressive symptoms (Roeser et al., 1998; Jia et al., 2009).

In the area of creativity, some theories have also suggested the beneficial role of student–student support. For example, Amabile (1996) in her componential theory of creativity proposed that under the climate of support within the peers such as student–student support, individuals perceive freedom and unrestriction. This, in turn, motivates them to engage in activities primarily for intrinsic interest and subsequently contributes to their development of creativity. Consistent with this theory, Deci and Ryan (1987) in their self-determination theory also claimed that individuals who perceive peer–peer support including student–student support feel a greater sense of autonomy, and with this, individuals are more willing to employ nontraditional approaches through which they want to come to decisions, thus enhancing creativity.

Although the theories mentioned above provide important insight into the link between student–student support and creativity, empirical evidence regarding the relationship between student–student support and creativity was scarce. To fill in this gap in the literature, the second purpose of the present study was to explore the effect of student–student support on junior high school students’ creative potential. On the study design, unlike the previous studies which only focused mainly on the between-person associations between predictors and creativity (Fürst et al., 2012; Meinel et al., 2018), the present study explored both the within-person and the between-person effects of student–student support on creative potential using multilevel linear modeling (within-person effects: the effects of within-person change in student–student support on concomitant within-person change in creative potential; between-person effects: the effect of student–student support on creative potential at the level of individual difference). The within-person and between-person design has several advantages over the pure between-person design in several ways. For example, it permits the exploration on the dynamic within-person associations between predictors and creative potential (Galla et al., 2014). From a methodological standpoint, it more guarantees the validity of the research conclusions compared with pure between-person designs. Specifically, unlike pure between-person designs, which are sensitive to unassessed variables that may confound the observed associations, within-person and between-person designs make it possible to methodologically control all time-stable (assessed and unassessed) between-person variables (e.g., gender) (Duckworth et al., 2010). Additionally, in pure between-person designs, the between-person and within-person effects can be confounded—because of not considering the nested structure of panel data—which yields an “uninterpretable blend” (Raudenbush and Bryk, 2002) of within-person and between-person effects. Within-person and between-person designs extend pure between-person designs by disaggregating the within-person and between-person effects (Enders and Tofighi, 2007; Duckworth et al., 2010), therefore improving the accuracy of the results.

### The Moderating Role of Need for Cognition in the Link Between Student–Student Support and Creative Potential

According to the componential theory of creativity (Amabile, 1996), similar school environments may have a different effect on creativity, depending on individuals’ personal attributes. Similarly, as Belsky and Pluess (2009) pointed out, personality characteristics may be factors that place individuals at various levels of susceptibility to the influence of both negative and positive environments. Thus, identifying personality characteristics that may relate to susceptibility is of particular importance to provide a more nuanced picture of the relation between student–student support and creative potential in junior high school students. Need for cognition—an individual’s tendency to join in and enjoy complex tasks (Cacioppo and Petty, 1982)—has been demonstrated to be an important “susceptible personality characteristic” to the effects of contextual factors on creativity across many studies (Osburn and Mumford, 2006; Nicholls, 2011; Madrid and Patterson, 2015). Therefore, this study sought to investigate the moderating role of need for cognition in the link between student–student support and creative potential.

Need for cognition enhances dedication and hardiness in thinking (Cacioppo and Petty, 1982) and serious consideration of accessible information (Levin et al., 2000). As such, while student–student support offers “expressive intellect,” need for cognition can be described based on tendencies for “controlled intellect” (Peabody and Goldberg, 1989). Creative ideas are characterized by both novelty and usefulness (Sternberg and Lubart, 1999; Plucker et al., 2004). An expressive intellect denoted by high student–student support should be tightly related to the generation of novel and nonconventional ideas (Pagano, 1979), whereas a controlled intellect offered by a high need for cognition might provide endurance and concentration to convert these nonconventional ideas into thoughts with higher levels of practicality and usefulness (Ivcevic and Mayer, 2007; Madrid and Patterson, 2015). In contrast, when need for cognition is low, individuals with high student–student support may generate novel and nonconventional thoughts with limited practicality and usefulness, due to the reduction of the effortful cognitive processing. In this regard, high levels of need for cognition might strengthen the beneficial effect of student–student support on creative potential.

### The Current Study

Regarding this research, three aims were established. First, the present study aimed to examine the developmental trend of creative potential in Chinese junior high school students. Second, the present study sought to explore the within-person and between-person effects of student–student support on creative potential. Third, the present study examined whether need for cognition moderates the relationship between student–student support and creative potential at the within-person and between-person levels.

We deduced that the aforementioned direct effects and moderation effects would be established for both within-person and between-person levels. The theoretical basis can be that within-person changes refer to temporal comparisons, which compare individuals’ present experiences to their prior ones and that between-person changes refer to social comparisons, which compare individuals’ experiences to other ones or to social norms (Curran and Bauer, 2011). It has been well acknowledged that individuals evaluate the meaning and significance of their experiences in both ways (Zell and Alicke, 2009; Strickhouser and Zell, 2015). Consequently, both positive within-person and between-person changes in student–student support and need for cognition could boost the self, thus resulting in positive effects in creative potential. Specifically, we hypothesized that (1) on the within-person level, time-varying student–student support would positively predict time-varying creative potential, and on the between-person level, average levels of student–student support positively predicted initial levels and developmental rates of creative potential; and (2) on the within-person level, high time-varying need for cognition would strengthen the beneficial effect of time-varying student–student support on time-varying creative potential, and on the between-person level, high average levels of need for cognition would strengthen the beneficial effects of average levels of student–student support on initial levels and developmental rates of creative potential. We did not hypothesize the developmental trend of creative potential in high school students, because of the lack of consistency on this issue in the previous literature.

## Materials and Methods

### Participants

Participants were recruited by the Longitudinal Study of Chinese Children and Adolescents. Two hundred and fourteen adolescents from six classes of two public junior high schools in a major metropolitan area of China participated in our survey. Students participated in our study during three consecutive school years (T1 = seventh grade, T2 = eighth grade, and T3 = ninth grade). The present study utilized data collected in 2012, 2013, and 2014. At T1, there were 214 students (46% girls, *M*_age_ = 13.29 years, *SD* = 0.49 years). Table 1 shows the family characteristics of the sample. From this table, it can be suggested that the sample was mostly middle class. At T2, a total of 209 participants were interviewed. Four participants transferred to another school, and one participant declined to continue to participate. At T3, a total of 188 participants were interviewed. Nineteen participants transferred to another school, and two participants did not consent. Participants’ participation at three time points is also shown in Table 2. During the 3 years, with the exception of very few students’ transferring in or out, the class members of each class remained unchanged. All participants’ parents signed written informed consent, and the current study received institutional review board approval from Shandong Normal University (Effects of Genes and Environment on Adolescents’ Creativity: A Longitudinal Study Based on Genome-Wide Study findings).

**TABLE 1:** Participants’ family characteristics at T1.

	**Father (*N*/total number)**	**Mother (*N*/total number)**
**Parents’ educational levels**		
College, bachelor, or above degree	61.2%	57.4%
High school or less degree	25.2%	29.0%
Missing	13.6%	13.6%
**Parents’ occupations**		
Professional, supervisory, or technological post	65.9%	58.8%
Working class	21.5%	27.6%
Missing	12.6%	13.6%
**Average household monthly income**		
Above ¥3,000	57%	
Below ¥3,000	28%	
Missing	15%	

*T1 = seventh grade.*

**TABLE 2:** Participants’ participation at three time points.

	**T1**	**T2**	**T3**
Participant number	214	209	188
Participation rate	100%	97.7%	87.9%

*T1 = seventh grade, T2 = eighth grade, and T3 = ninth grade.*

To examine whether the loss of the participants was associated with the major variables we focused on, we compared participants accomplishing versus those not accomplishing all three time points. The *t*-tests indicated that there were no group differences on age [*t*(212) = 1.09, *p* > 0.05]; on creative potential [fluency: *t*(212) = 0.52, *p* > 0.05, flexibility: *t*(212) = 1.14, *p* > 0.05, originality: *t*(212) = −0.34, *p* > 0.05]; on student–student support [*t* (212) = 0.24, *p* > 0.05]; or on need for cognition [*t*(212) = 0.67, *p* > 0.05]. Chi-square tests revealed that there existed no group difference on gender, *χ*^2^(1) = 0.15, *p* > 0.05. Collectively, these results suggested that the influence of the missing values was minimal.

Since missing data were proven to be missing randomly, we adopted the EM procedure to pad the missing indicator item values (Schafer and Graham, 2002). Compared with other means, for example, listwise deletion, this procedure generates less deviant and more valid results (Schafer and Graham, 2002; Baraldi and Enders, 2010).

### Measures

#### Creative Potential

Creative potential was measured using the Real-life Problem Solving Test, which is embedded in the Creativity Assessment Battery, developed by Runco (rCAB; Creativity Testing Service, Bishop, GA, United States). The participants were offered one of three parallel versions of the Real-life Problem Solving Test (Versions A–C) at each wave. Each version contains three tasks, and each task involves an open-minded real-life problem. Participants were required to write solutions. The requirement is that the more solutions, the better and that the more creative the solutions, the better. An example of the tasks is as follows: “You have a neighbor of your own age who talks a lot and never thinks about other people’s feelings. One Saturday, you are reading a book you like very much. At this moment, someone knocks at the door. You know he is standing outside the door. If you can come up with some solutions to get rid of him, you can continue to enjoy reading.” Each task was given 4 min. We rated the fluency, flexibility, and originality of each task. Fluency refers to individuals’ abilities to generate numerous ideas; flexibility refers to individuals’ abilities to generate ideas for several conceptual categories; originality refers to individuals’ abilities to generate novel ideas. To rate the three indexes, the solutions which are not useful and appropriate were first eliminated. Next, fluency was scored by summing the number of the answers generated by every participant. To calculate flexibility, three judges (two of them were doctoral students and the other one was a graduate student, and all of them majored in psychology and studied creativity in China) first jointly discussed and determined 8 to 10 different conceptual categories based on the specific content of participants’ responses. They then separately allocated all participants’ responses to the categories and separately calculated each participant’s flexibility score by adding up the number of different categories used in one participant’s responses. The participant’s final flexibility score was obtained by averaging the three judges’ scores (the interrater reliabilities for all the tasks were higher than 0.95). Originality was obtained by adding up the number of original answers (answers generated by no more than 5% of the total number of the participants). Previous studies employing this measurement tool among Chinese adolescents have been demonstrated to have good reliability and validity (Ren et al., 2017). In the present study, at T1, Cronbach’s αs of fluency, flexibility, and originality were 0.72, 0.74, and 0.81, respectively. At T2, Cronbach’s αs of fluency, flexibility, and originality were 0.81, 0.80, and 0.85, respectively. At T3, Cronbach’s αs of fluency, flexibility, and originality were 0.77, 0.82, and 0.73, respectively.

#### Student–Student Support

Student–student support was measured using the Chinese adaption (Jia et al., 2009) of the student–student support subscale from the perceived school climate scale (Emmons et al., 2002; Brand et al., 2003). The student–student support subscale contains 13 items, for example, “students care about each other,” “students fight a lot,” and “students trust each other” (1: never to 4: always). Student–student support was obtained by averaging the 13th items, and the higher the score was, the higher an individual perceives student–student support. Previous studies employing this scale among Chinese samples have high internal consistency, and this scale has been validated to be used with adolescents as well (Jia et al., 2009). In the present study, at T1, Cronbach’s α was 0.91; at T2, Cronbach’s α was 0.91; and at T3, Cronbach’s α was 0.88. To ensure the validity of this scale, a confirmatory factor analysis (CFA) was conducted in AMOS 7.0. The results of the CFA suggested a superb fit (T1, T2, and T3; *χ*^2^/*df* = 2.3, 2.6, and 3.1; *GFI* = 0.91, 0.93, and 0.97; *CFI* = 0.94, 0.96, and 0.98; *IFI* = 0.95, 0.96, and 0.98; *NFI* = 0.91, 0.94, and 0.97; and *RMSEA* = 0.08, 0.09, and 0.10).

#### Need for Cognition

Need for cognition was assessed using the Chinese adaption (Gao, 1994) of the need for cognition scale (Cacioppo and Petty, 1982). The scale contains 18 items, for example, “the idea that relying on thinking makes you the best person attracts me a lot,” “thinking about abstract problems appeals to me,” and “I really enjoy tasks that involve thinking up new ways to solve problems” (1: completely disagree to 5: completely agree). Need for cognition was calculated by taking the mean of the 18 items, with higher scores suggesting higher levels of need for cognition. This scale has been demonstrated to have high reliability and validity among Chinese adolescents (Yang and Zhang, 2004; Shi and Xu, 2008). In the present study, at T1, Cronbach’s α was 0.87; at T2, Cronbach’s α was 0.88; and at T3, Cronbach’s α was 0.87. A CFA suggested a superb fit (T1, T2, and T3; *χ*^2^/*df* = 1.5, 1.8, and 2.3; *GFI* = 0.92, 0.93, and 0.92; *CFI* = 0.96, 0.97, and 0.94; *IFI* = 0.96, 0.97, and 0.94; *NFI* = 0.90, 0.94, and 0.90; *RMSEA* = 0.05, 0.06, and 0.08).

#### Academic Performance

In the present study, participants’ final examination scores at T1 were collected, including Chinese, math, English, politics, history, geography, and biology scores. Each student’s academic performance was scored by first transforming the seven subjects’ scores to *z*-scores and then adding them up.

### Procedure

We adopted the method of random cluster sampling to sample two middle schools and sent the two schools invitation letters via email which describe the purpose and procedures of our study. Both of the schools responded and agreed to participate in this study. In each school, three classes in grade seven were randomly selected. Of the participating students, informed consent of their parents as well as their own informed assent was obtained before the start of the study. During the study, students were asked to complete several questionnaires when a regular class began, which lasted for 40 min. At each wave, these questionnaires were presented in the same order, and each participant received a small gift for their participation. All procedures used in the present study containing participants were in accordance with the ethical standards of the ethics committee on human experimentation and with the 1964 Helsinki declaration and its later amendments or comparable ethical standards.

### Analytic Plan

Because within-person level data were nested within between-person level data, HLM (Raudenbush and Bryk, 2002) was run to test the hypotheses. In order to reduce multicollinearity, all predictor variables at level 1 were group centered, and all predictor variables concerning the relationship at level 2 were grand centered (Aiken and West, 1991). Additionally, we included academic performance as the control variable in all conditional models, since academic performance was demonstrated to have a positive stable correlation with creativity (Gajda et al., 2017). Specifically, four steps were taken to test the hypotheses. First, we ran a set of null models which no predictors were in to examine whether there exists between-person variation on creative potential (fluency, flexibility, and originality). Second, we conducted a series of multilevel models with two levels to examine the initial level and the developmental rate of creative potential. Third, we examined whether student–student support promotes creative potential at the within-person and between-person levels. Fourth, we explored whether need for cognition moderates the link between student–student support and creative potential at the within-person and between-person levels. A sample full model in which fluency was the independent variable was presented here:

Level 1 (the within-person level)

$${\text{Fluency}}_{i\,j}=\beta_{0\,j}+\beta_{1\,j}\,\left(\mathrm{time}\right)+\beta_{2\,j}\,\left(\mathrm{student}-\mathrm{student}\,\mathrm{support}\right)+\beta_{3\,j}\left(\mathrm{need}\mathrm{for}\mathrm{cognition}\right)+\beta_{4\,j}(\mathrm{the}\mathrm{interaction}\mathrm{between}\mathrm{student}-\mathrm{student}\mathrm{support}\mathrm{and}\mathrm{need}\mathrm{for}\mathrm{cognition})+e{}_{\mathrm{ij}}$$

Level 2 (the between-person level)

$$\beta_{0\,j}=\gamma_{00}+\gamma_{01}\left(\mathrm{academic}\mathrm{performance}\right)+\gamma_{02}(\mathrm{average}\mathrm{student}-\mathrm{student}\mathrm{support})+\gamma {}_{03}(\mathrm{average}\mathrm{need}\mathrm{for}\mathrm{cognition})+\gamma {}_{04}(\mathrm{the}\mathrm{interaction}\,\mathrm{between}\,\mathrm{average}\,\mathrm{student}-\mathrm{student}\,\mathrm{support}\,\mathrm{and}\mathrm{average}\mathrm{need}\,\mathrm{for}\,\mathrm{cognition})+u{}_{0\,j}$$

$$\beta_{1\,j}=\gamma_{10}+\gamma_{11}\left(\mathrm{academic}\mathrm{performance}\right)+\gamma_{12}(\mathrm{average}\mathrm{student}-\mathrm{student}\mathrm{support})+\gamma {}_{13}(\mathrm{average}\mathrm{need}\mathrm{for}\mathrm{cognition})+\gamma {}_{14}(\mathrm{the}\mathrm{interaction}\,\mathrm{between}\,\mathrm{average}\,\mathrm{student}-\mathrm{student}\,\mathrm{support and}\mathrm{average}\,\mathrm{need}\,\mathrm{for}\,\mathrm{cognition})+u{}_{1\,j}$$

$$\beta_{2\,j}=\gamma_{20}$$

$$\beta_{3\,j}=\gamma_{30}$$

$$\beta_{4\,j}=\gamma_{40}$$

## Results

### Descriptive Statistics

Table 3 shows means, standard deviations, and zero-order correlations for primary variables. T1 academic performance was positively correlated with fluency, flexibility, and originality at all data waves, except T2 originality. T1 student–student support was positively correlated with fluency, flexibility, and originality at T1 but was not correlated with those at T2 or T3. T2 student–student support was positively correlated with fluency, flexibility, and originality from T1 through T2 but was not correlated with those at T3. T3 student–student support was positively correlated with fluency, flexibility, and originality at T3 but was not correlated with those at T1 or T2. Both T1 and T2 need for cognition were positively correlated with fluency, flexibility, and originality at all three waves except T3 originality. T3 need for cognition was positively correlated with fluency, flexibility, and originality at T3 but was not correlated with those at T1 or T2. Student–student support was positively correlated with need for cognition at all time points.

**TABLE 3:** Descriptive statistics and correlations between academic performance, student–student support, need for cognition, and creative potential.

	***M***	***SD***	**1**	**2**	**3**	**4**	**5**	**6**	**7**	**8**	**9**	**10**	**11**	**12**	**13**	**14**	**15**	**16**
1 T1 Ap	0	5.96																
2 T1 Sss	3.26	0.48	0.01															
3 T1 Nc	3.55	0.67	0.26**	0.35**														
4 T1 Fluency	5.96	1.99	0.23**	0.15*	0.30**													
5 T1 Flexibility	3.70	0.96	0.22**	0.14*	0.25**	0.74**												
6 T1 Originality	1.89	1.09	0.18**	0.19**	0.32**	0.80**	0.58**											
7 T2 Sss	3.30	0.46	0.05	0.49**	0.28**	0.20**	0.18*	0.22**										
8 T2 Nc	3.53	0.69	0.21**	0.28**	0.57**	0.22**	0.23**	0.24**	0.35**									
9 T2 Fluency	5.50	3.55	0.15*	0.12	0.28**	0.51**	0.40**	0.49**	0.23**	0.35**								
10 T2 Flexibility	3.47	2.40	0.14*	0.13	0.27**	0.48**	0.39**	0.48**	0.24**	0.32**	0.95**							
11 T2 Originality	1.76	1.57	0.11	0.09	0.29**	0.45**	0.36**	0.48**	0.25**	0.33**	0.86**	0.83**						
12 T3 Sss	3.21	0.45	–0.02	0.35**	0.14*	0.08	0.06	0.08	0.55**	0.20**	0.05	0.03	0.03					
13 T3 Nc	3.35	0.60	0.18**	0.21**	0.40**	–0.02	–0.01	0.01	0.17*	0.40**	0.03	–0.01	–0.03	0.33**				
14 T3 Fluency	5.04	2.52	0.30**	–0.01	0.18**	0.33**	0.31**	0.26**	0.11	0.23**	0.37**	0.35**	0.27**	0.20**	0.26**			
15 T3 Flexibility	3.41	1.21	0.29**	–0.04	0.15*	0.26**	0.28**	0.19**	0.08	0.15*	0.20**	0.19**	0.11	0.18**	0.27**	0.88**		
16 T3 Originality	0.34	0.40	0.14*	–0.04	0.08	0.12	0.05	0.14*	0.09	0.12	0.24**	0.25**	0.27**	0.15*	0.23**	0.55**	0.51**	

*p ¡ 0.05, **p < 0.01. T1 = 2012 year, T2 = 2013 year, and T3 = 2014 year. Ap, academic performance; Sss, student–student support; Nc, need for cognition. Missing data were padded using the EM procedure.

### Unconditional Means Models

We adopted the HLM procedure (Hofmann, 1997) to test our hypotheses. In order to test our hypotheses, there should exist between-person variance in fluency, flexibility, and originality. A chi-square test revealed that the between-person variance in fluency, flexibility, and originality was significant [fluency: *χ*_(213)_^2^/df = 2.70, *p* < 0.001; flexibility: *χ*_(213)_^2^/df = 1.82, *p* < 0.001; originality: *χ*_(213)_^2^/df = 1.19, *p* < 0.05]. Based on these results from testing the unconditional means models, the next analyses were performed.

### The Initial Level and the Developmental Rate of Creative Potential

To explore the initial level and the developmental rate of creative potential, three models (models 1, 4, and 7) were fitted: in which fluency, flexibility, and originality served as the dependent variables, respectively; time was the level 1 predictor, and the intercept coefficients and the slope (time) coefficients obtained from level 1 were regressed at level 2. The results revealed that the intercepts were significant on fluency (*I* = 5.96, *t* = 34.93, *p* < 0.001), flexibility (*I* = 3.67, *t* = 41.66, *p* < 0.001), and originality (*I* = 2.11, *t* = 25.02, *p* < 0.001), suggesting that the initial levels of fluency, flexibility, and originality were significantly greater than zero. The results also revealed that the slopes were significant on fluency (*S* = −0.46, *t* = −5.11, *p* < 0.001), flexibility (*S* = −0.15, *t* = −3.25, *p* < 0.001), and originality (*S* = −0.78, *t* = −20.54, *p* < 0.001), suggesting that there existed downward trends on fluency, flexibility, and originality see Table 4.

**TABLE 4:** Testing the within-person and between-person effects of student–student support on creative potential and the moderating role of need for cognition on the link between student–student support and creative potential.

	**Fluency**			**Flexibility**			**Originality**		
	**Model 1**	**Model 2**	**Model 3**	**Model 4**	**Model 5**	**Model 6**	**Model 7**	**Model 8**	**Model 9**
Fixed effects									
Intercept	5.96***	5.84***	5.84***	3.67***	3.60***	3.58***	2.11***	2.05***	2.04***
Ap		0.07***	0.07***		—	—		0.01+	0.01+
Average Sss		0.57	0.58		—	—		0.17	0.19
Average Nc		0.93***	0.92***		—	—		0.35***	0.31***
Average Sss × Average Nc			—			—			—
Slope	−0.46***	−0.34***	−0.34***	−0.15***	–0.07	−0.08+	−0.78***	−0.72***	−0.72***
Ap		—	—		—	—		—	—
Average Sss		—	—		—	—		—	—
Average Nc		—	—		—	—		—	—
Average Sss × Average Nc		—	—		—	—		—	—
Time-varying Sss		0.83**	0.84**		0.56**	0.59**		0.49***	0.53***
Time-varying Nc		1.00***	1.01***		0.58***	0.59***		0.47***	0.49***
Time-varying Sss × Time-varying Nc			0.32			0.88*			0.68*
Random effects									
Intercept	2.31***	1.85**	1.85**	0.55	0.53	0.54	0.87***	0.69***	0.70***
Slope	0.04	0.03	0.03	0.01	0.01	0.01	0.10	0.07	0.08
Level 1 residual	4.71	4.37	4.38	2.12	1.99	1.98	0.92	0.86	0.85

+p < 0.1*, p < 0.05, **p < 0.01, ***p < 0.001. Ap, academic performance; Sss, student-student support; Nc, need for cognition. “—” Represents the effect of independent variable on dependent variable was not be estimated. The parameters of fixed effects are unstandardized regression coefficients. The parameters of random effects are estimates of variance. Missing data were padded using the EM procedure.

On the between-person levels, there existed between-person variation on the initial level of fluency (*σ*^2^ = 2.31, *χ*^2^ = 297.53, *p* < 0.001) and originality (*σ*^2^ = 0.87, *χ*^2^ = 425.63, *p* < 0.001), but not flexibility (*σ*^2^ = 0.55, *χ*^2^ = 201.29, *p* > 0.05). The results also revealed that there did not exist between-person variation on the developmental rate of fluency (*σ*^2^ = 0.04, *χ*^2^ = 157.33, *p* > 0.05), flexibility (*σ*^2^ = 0.01, *χ*^2^ = 87.83, *p* > 0.05), or originality (*σ*^2^ = 0.10, *χ*^2^ = 142.55, *p* > 0.05). See Table 4. Given that there was only between-person variation on the initial level of fluency and originality, in the following analyses, we only examined between-person effects of student–student support on the initial level of fluency and originality.

### Within-Person and Between-Person Effects of Student–Student Support on Creative Potential

To explore within-person and between-person effects of student–student support on creative potential, three models (models 2, 5, and 8) were fitted: in which fluency, flexibility, and originality served as the dependent variables, respectively; time, student–student support, and need for cognition were the level 1 predictors; average student–student support, average need for cognition, and academic achievement (the control variable) were the level 2 predictors. Results are shown in Table 4. At the within-person level, time-varying student–student support positively predicted time-varying fluency (*B* = 0.83, *t* = 2.80, *p <* 0.01), time-varying flexibility (*B* = 0.56, *t* = 2.87, *p* < 0.01), and time-varying originality (*B* = 0.49, *t* = 3.66, *p* < 0.001). At the between-person level, average student–student support did not significantly predict the initial level of fluency (*B* = 0.57, *t* = 1.28, *p >* 0.05) or the initial level of originality (*B* = 0.17, *t* = 1.14, *p* > 0.05). Because the results suggested that there were no between-person effects of student–student support on fluency or originality, in the following analyses, we did not explore the moderating roles of need for cognition in the links between student–student support and fluency or originality at the between-person level.

### The Moderating Role of Need for Cognition on the Link Between Student–Student Support and Creative Potential at the Within-Person Level

On the basis of models (models 2, 5, and 8), we added the interaction of student–student support and need for cognition to level 1 of models 2, 5, and 8, forming models 3, 6, and 9, respectively. Results are shown in Table 4. At the within-person level, time-varying need for cognition did not moderate the effect of time-varying student–student support on time-varying fluency (*B* = 0.32, *t* = 0.58, *p* > 0.05). However, time-varying need for cognition moderated the effects of time-varying student–student support on time-varying flexibility (*B* = 0.88, *t* = 2.20, *p* < 0.05) and time-varying originality (*B* = 0.68, *t* = 2.23, *p* < 0.05).

**FIGURE 1:**
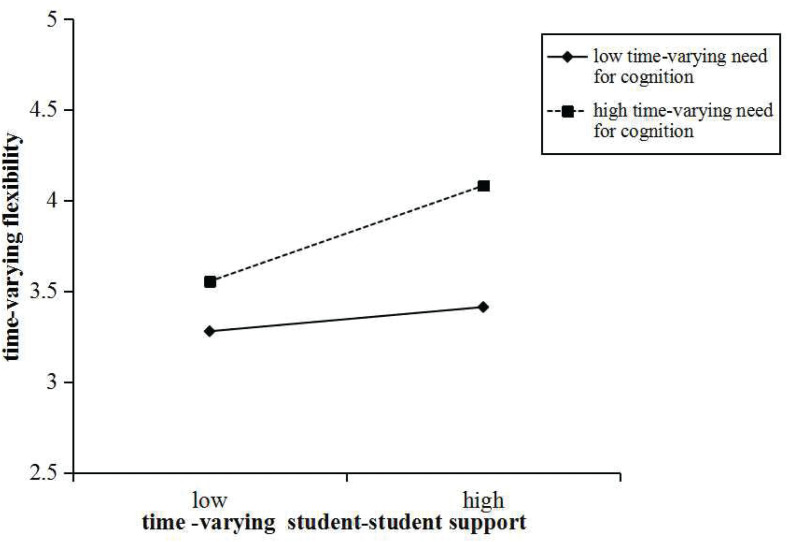
Plot of the within-person moderating effect of time-varying need for cognition on the relationship between time-varying student–student support and time-varying flexibility.

**FIGURE 2:**
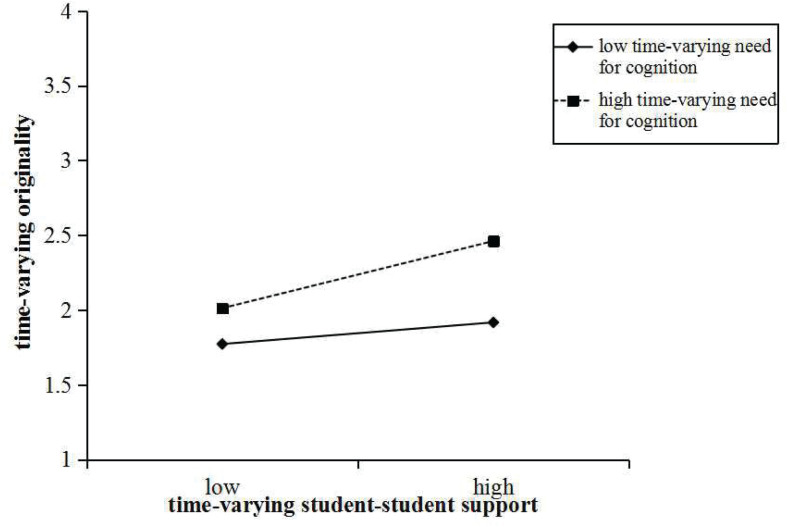
Plot of the within-person moderating effect of time-varying need for cognition on the relationship between time-varying student–student support and time-varying originality.

We then tested the two significant within-person moderation effects utilizing a simple slope test (Preacher et al., 2006). The interaction plots (Figures 1 and 2) graphically represent the within-person moderation, showing the relationships between time-varying student–student support and time-varying flexibility and time-varying originality, respectively, with (1) high and (2) low time-varying need for cognition. As expected, time-varying student–student support had stronger associations with time-varying flexibility and time-varying originality, respectively, when time-varying need for cognition was in a high level (+1 SD; flexibility: *b* = 0.94, *t* = 3.15, *p* < 0.01; originality: *b* = 0.80, *t* = 3.33, *p* < 0.001) rather than in a low level (−1 SD; flexibility: *b* = 0.24, *t* = 1.09, *p* > 0.05; originality: *b* = 0.26, *t* = 1.93, *p* > 0.05). Briefly, the interaction plots show that time-varying need for cognition intensified the positive relationships between time-varying student–student support and time-varying flexibility and time-varying originality, respectively.

## Discussion

The current research was designed to shed light on the question of what the developmental trend of creative potential in Chinese junior high school students is and how student–student support and need for cognition affect creative potential. It had several strengths in solving these questions. First, by using longitudinal multilevel linear models, it is possible to describe the real developmental trend of creative potential of Chinese junior high school students, moving beyond cross-sectional studies from which only inferential claims of adolescents’ developmental trend can be made. Second, the present study systematically explored not only the stable between-person associations among student–student support, need for cognition, and creative potential but also the dynamic within-person associations among these variables, allowing for a more fine-grained picture of the relationships between student–student support, need for cognition, and creative potential. Third, by using between-person and within-person designs, the validity of the research results can be more ensured, because it addressed several important methodological shortcomings of pure between-person designs such as not thoroughly separating the within-person and the between-person effects and not methodologically controlling for time-invariant characteristics (Duckworth et al., 2010; Galla et al., 2014).

### Developmental Trends of Chinese Junior High School Students’ Creative Potential

The present study found that creative potential showed a downward trend from grades 7 to 9 in Chinese junior high school students. The result was consistent with an earlier longitudinal finding obtained by Camp (1994). Moreover, it also accorded with a cross-sectional finding observed in Mainland China (Yi et al., 2013). This downward trend may be due to several reasons. First, junior high school students are characterized by highly developed self-consciousness and subjective bigotry (Wang and Holcombe, 2010). As a result, they are often self-righteous, reject others’ suggestions, and are sensitive to others’ evaluation, which is not conducive to their objective judgments of things and further restricts the development of their creative potential (Zhang and Gu, 2004). Second, for students, entering junior high schools from primary schools marks the beginning of adolescence (Erikson, 1966). During this period, junior high school students tend to have more social comparison and pay special attention to external competition (Eccles et al., 1984; Rosenholtz and Simpson, 1984), so that they might not well focus on the innovative activities they are engaged in. Third, China’s high valuing of college entrance exams raises great academic pressure in junior high school students, which seriously stifles their creative potential (Yi et al., 2013). Fourth, unlike primary schools of China in which quality education is highly pursued, education of middle schools in China is highly exam oriented. Thus, teachers and students in middle schools often put more emphasis on uniform and standard answers of tests (Niu and Sternberg, 2001). This, in turn, greatly hinders the healthy development of middle school students’ creative potential (Gu, 2013).

### Association Between Student–Student Support and Creative Potential

Regarding the effect of student–student support on Chinese junior high school students’ creative potential, we found a dynamic relationship between student–student support and creative potential at the within-person level. That is, time-varying student–student support positively predicted time-varying creative potential (fluency, flexibility, and originality). In other words, between grades 7 and 9, when a student experienced increased student–student support, one’s creative potential also increased accordingly. The results confirmed the theoretical expectations of the componential theory of creativity (Amabile, 1996), which postulated that peer–peer support such as student–student support supplies a free and unlimited climate, so that adolescents are able to take part in activities out of their own interest rather than external conditions, which promotes their creativity. In addition, this result can also be accounted for by another theory: the self-determination theory (SDT; Deci and Ryan, 1987). The theory stated that under the climate of peer–peer support including student–student support, students can act with a full sense of choice and volition. This, in turn, enables them to use nontraditional and novel approaches they like to reach decisions, thus enhancing creativity.

At the between-person level, however, we found a different conclusion that the average level of student–student support did not significantly predict the initial level and the developmental rate of creative potential (fluency, flexibility, and originality). One possible explanation is that between-person changes denote social comparisons, which involve a comparison with others in one’s environment (Curran and Bauer, 2011). Therefore, adolescents’ high student–student support at between-person levels might be “publicly” known by classmates. This might, to some extent, result in adolescents’ feeling peer pressure, which would not benefit their creative potential. The different results further demonstrated the significance of incorporating analytic methods which were able to disaggregate the within-person and between-person effects of student–student support on creative potential.

### The Moderating Role of Need for Cognition

Regarding the moderating role of need for cognition, we also found the within-person effect. That is, time-varying need for cognition moderated the positive effects of time-varying student–student support on time-varying flexibility and time-varying originality. In other words, between grades 7 and 9, when a student experienced a high level of increase in need for cognition, increased student–student support predicted greater increase in flexibility and originality. Flexibility refers to an individual’s ability to think flexibly and to get rid of thinking stereotypes (Butler et al., 2003). As Runco (2014) pointed out, due to stubbornness of individuals’ mindset, more will efforts can help individuals get rid of these ingrained stereotypes more easily and thus contribute to the generation of more flexible thoughts. Because high levels of need for cognition ensure that individuals continue to put in a large number of will efforts (Madrid and Patterson, 2015), high need for cognition could enhance individuals who perceive high levels of student–student support to think more flexibly. On the other hand, originality represents the quality of the generated ideas, and these ideas are truly creative thoughts characterized by both novelty and usefulness (Dixon, 1979; Mcvearry et al., 2009). As we stated in the introduction, high levels of student–student support, as an expressive intellect, could be tightly related to the generation of nonconventional and novel ideas (Pagano, 1979). However, these nonconventional and novel ideas are not necessarily ideas of usefulness and practicality. High levels of need for cognition, as a controlled intellect, could play a role in enhancing the usefulness and practicality of these nonconventional and novel ideas by offering endurance and concentration (Ivcevic and Mayer, 2007; Madrid and Patterson, 2015). Thus, need for cognition could strengthen the beneficial effect of student–student support on originality.

Our results about the moderating role of need for cognition in the beneficial effect of student–student support on creative potential confirmed the theoretical expectations of dual models of creativity (George, 2011) which highlighted the needs for both expressive and controlled psychological processes in creative behaviors. In addition, it is also congruent with alternative research streams of research (George and Zhou, 2007; Baas et al., 2008; De Dreu et al., 2008), which suggested that creativity not only requires ideation for the emergence of novel thoughts but also requires mental persistence to reach useful creative solutions.

### Limitations and Future Directions

The present work is not without limitations that should be addressed in future studies. First, the generalizability of the current findings is somewhat limited because the current sample only comprised Chinese junior high school students. Chinese junior high school students have more academic pressure, whereas Western adolescents own a more free educational environment (Niu, 2007; Zhang et al., 2018). As a result, there may be qualitative differences in the developmental trend of creative potential between junior high school students in China and those in Western countries. In addition to this potential culture difference in the developmental trend of creative potential, because adolescents might attach a different interpretation and affective connotation to high consistency among classmates between Western and Eastern cultures, the relationships between student–student support and creative potential may also be culturally different. Thus, cross-cultural research is required. Second, creative potential can be reflected by a series of indicators such as creative thinking (e.g., divergent thinking and convergent thinking), creative personality, creative achievement, and many other components. However, the present study only employed divergent thinking ability to capture creative potential. Future research should retest the relationships between student–student support, need for cognition, and creative potential using other creativity tests. Finally, the sample was from middle-class families. Therefore, we should take caution in generalizing these observed findings before they can be verified across all kinds of families.

## Conclusion

The present research adds to our understanding of the systemic links between student–student support, need for cognition, and creative potential. We determined three valuable conclusions. First, Chinese junior high school students’ creative potential showed a downward trend from grades 7 to 9. Second, at the within-person level, time-varying student–student support positively predicted time-varying creative potential. Third, at the within-person level, time-varying need for cognition moderated the positive link between time-varying student–student support and time-varying creative potential. Fourth, at the between-person level, no support was found for the links between student–student support, need for cognition, and creative potential. Specifically, average levels of student–student support neither significantly predicted initial levels and developmental rates of creative potential nor moderated the links between average levels of student–student and initial levels and developmental rates of creative potential.

## Data Availability Statement

The datasets using in this study are available from the corresponding author on reasonable request.

## Ethics Statement

The studies involving human participants were reviewed and approved by Institutional Review Board of Shandong Normal University. Written informed consent to participate in this study was provided by the participants’ legal guardian/next of kin.

## Author Contributions

PC: conceptualization, methodology, roles/writing – original draft, and writing – review. JZ: conceptualization, funding acquisition, supervision, and writing – review. Both authors contributed to the article and approved the submitted version.

## Conflict of Interest

The authors declare that the research was conducted in the absence of any commercial or financial relationships that could be construed as a potential conflict of interest.
